# Non-HDL Cholesterol and Residual Cardiometabolic Risk in Middle Eastern Patients with Atherosclerotic Cardiovascular Disease

**DOI:** 10.3390/healthcare14050565

**Published:** 2026-02-25

**Authors:** Osama Alkouri, Ahmad M. Al-Bashaireh, Alanoud Alobaidly, Ghaleb Alharbi, Rahma Musaed Alabkal, Abdullah Hasan, Ayman Hammoudeh, Nisreen Nusair, Jolly Isaac, Abdulkareem Alshehri, Ahmad Rajeh Saifan, Nezam Al-Nsair

**Affiliations:** 1 Faculty of Nursing, Yarmouk University, P.O. Box 566, Irbid 21163, Jordan; o.alkouri@yu.edu.jo (O.A.); ahmad.saifan@yu.edu.jo (A.R.S.); 2 Faculty of Nursing, Philadelphia University, Jarash Road, Amman 19392, Jordan; aalbashaireh@gmail.com; 3 College of Nursing and Health Sciences, Flinders University, Bedford Park, SA 5042, Australia; alanoud.alobaidly@flinders.edu.au; 4 Department of Clinical Pharmacy, College of Pharmacy, Shaqra University, Shaqra 11961, Saudi Arabia; g.alharbi@su.edu.sa; 5 Department of Pharmacy, Ministry of Health, Kuwait City 13001, Kuwait; ralabkal@hotmail.com; 6 College of Nursing, The Public Authority for Applied Education and Training, Shuwaikh, Kuwait City 72853, Kuwait; ay.hasan@paaet.edu.kw; 7 Department of Cardiology, Istishari Hospital, Amman 11184, Jordan; hammoudeh_ayman@yahoo.com; 8 Department of Chemistry and Biochemistry, Miami University, Oxford, OH 45056, USA; nusairn1@miamioh.edu; 9 Hind Bint Maktoum College of Nursing and Midwifery, Mohammed Bin Rashid University of Medicine and Health Sciences, Dubai Health, Dubai Health Care City, Dubai 505055, United Arab Emirates; jolly.isaac@dubaihealth.ae; 10 Advanced Diagnostic and Therapeutic Institute, King Abdulaziz City for Science and Technology (KACST), Riyadh 12354, Saudi Arabia; abalshehri@kacst.gov.sa

**Keywords:** atherosclerotic cardiovascular disease, non-HDL cholesterol, residual cardiovascular risk, lipid ratios, Middle East

## Abstract

**Background**: Although low-density-lipoprotein cholesterol (LDL-C) remains the primary target of lipid-lowering therapy, many patients with atherosclerotic cardiovascular disease (ASCVD) continue to experience cardiovascular events. This residual risk suggests that LDL-C alone does not fully capture the total atherogenic burden. Alternative lipid measures, including non-high-density lipoprotein cholesterol (non-HDL-C) and cholesterol ratios, may provide a more comprehensive risk assessment, particularly in populations with a high prevalence of metabolic disorders. This study assessed the prevalence and clinical determinants of elevated non-HDL cholesterol and adverse cholesterol ratios among Middle Eastern patients with established ASCVD. **Methods**: This pooled cross-sectional analysis included 2763 adults with confirmed ASCVD from the Jordan SMuRF-less Study and six cardiovascular registries across the Middle East. Patients were stratified by standard modifiable cardiovascular risk factors (SMuRFs). Demographic, clinical, treatment, and lifestyle data were harmonized and analyzed using multivariable regression models. **Results**: Elevated non-HDL-C was observed in 54% of patients. Those with elevated non-HDL-C were younger (55.0 vs. 59.9 years) and more frequently current smokers (52.6% vs. 43.1%). Hypertension (64.2% vs. 51.0%) and heart failure (25.0% vs. 15.4%) were more common among patients with lower non-HDL-C, whereas dyslipidemia (90.8% vs. 75.8%) and acute coronary syndrome (88.1% vs. 83.7%) were more prevalent in the elevated group. Elevated non-HDL-C was associated with higher baseline LDL-C levels measured prior to the initiation of lipid-lowering therapy (141.3 vs. 81.1 mg/dL) and higher triglycerides (221.4 vs. 140.9 mg/dL). In multivariable analyses, age > 60 years (OR = 0.45), hypertension (OR = 0.74), and heart failure (OR = 0.61) were inversely associated with elevated non-HDL-C. **Conclusions**: Elevated non-HDL cholesterol is common among Middle Eastern patients with ASCVD, particularly younger individuals, reflecting early metabolic risk and increased atherogenic burden. Non-HDL-C is a valuable marker of residual cardiovascular risk, supporting earlier screening and region-specific prevention strategies.

## 1. Background

Atherosclerotic cardiovascular disease (ASCVD) remains a leading global cause of morbidity and mortality, with its burden rising in the Middle East due to increasing modifiable risk factors [1,2]. While low-density-lipoprotein cholesterol (LDL-C) has traditionally been the primary target in lipid-lowering therapies [3,4], growing evidence suggests that it does not fully capture the spectrum of atherogenic lipoproteins associated with cardiovascular risk [5]. Many individuals achieving LDL-C targets still experience events, indicating residual risk beyond LDL-C [5]. This has spurred interest in alternative markers such as non-high-density lipoprotein cholesterol (non-HDL-C) and cholesterol ratios, which may offer a more comprehensive assessment of atherogenic burden [6].

Non-HDL-C, calculated as total cholesterol minus HDL-C, encompasses all atherogenic particles, including LDL-C, VLDL, IDL, Lp (a), and chylomicron remnants, which contribute to plaque formation [6]. It is simple to compute, does not require fasting, and has been proposed as a standalone marker for diagnosing and managing atherosclerosis, especially in dyslipidemic patients [7,8]. Similarly, the total cholesterol-to-HDL-C (TC/HDL-C) ratio reflects the balance between harmful and protective lipids. A ratio < 3.5 indicates lower risk, whereas a ratio > 5.0 suggests higher risk, providing a more nuanced profile than individual lipid values [9,10].

Major guidelines, including those from the American Association of Clinical Endocrinology and the European Society of Cardiology, recommend non-HDL-C as a secondary treatment target, particularly in patients with diabetes or high triglycerides [11]. A low non-HDL cholesterol level of <130 mg/dL is common, although some recommend it to be ≤30 mg/dL above the LDL-C goal (e.g., <100 mg/dL if the LDL-C goal is <70 mg/dL) [12]. However, emerging data challenge the universal applicability of this threshold, highlighting the need for context-specific interpretation based on ethnicity, metabolic profile, and regional trends [6].

Despite global support for non-HDL-C and cholesterol ratios, data from the Middle East remain scarce [6]. Genetic, dietary, and lifestyle factors unique to the region affect cardiovascular risk, but most studies are based on Western or East Asian populations [6]. No major study has yet assessed non-HDL-C and cholesterol ratios in Middle Eastern ASCVD patients, despite the region’s high prevalence of dyslipidemia, diabetes, tobacco use, and inactivity, often manifesting at younger ages [13,14,15]. Moreover, disparities in healthcare access and treatment may influence lipid outcomes [13].

This study is among the first large-scale analyses of non-HDL-C and cholesterol ratios in Middle Eastern populations with a history of ASCVD. Drawing from the prospective Jordan SMuRF-less Study and six regional cardiovascular registries, patients were categorized by non-HDL-C levels (<130 vs. ≥130 mg/dL) and TC/HDL-C ratios (<2, 2–5, >5). The analysis explored associations with demographics, comorbidities, treatment patterns, and residual risk among patients who met LDL-C targets. We also examined age- and sex-based differences, treatment trends, and the potential of non-HDL-C and cholesterol ratios to better reflect residual cardiovascular risk than LDL-C alone. Given the region’s high prevalence of metabolic disease, we hypothesize these markers may offer superior risk stratification. This study highlights the limitations of relying solely on LDL-C. It underscores the need for region-specific prevention strategies and lipid targets to improve outcomes in this high-risk, underserved population.

## 2. Materials and Methods

### 2.1. Study Design

This study utilized cross-sectional design from different resources; therefore, findings should be interpreted with caution, acknowledging the potential for survival bias and reverse causation. This study utilized data derived from two primary sources. The first study was a prospective cohort comprising individuals aged 18 years and older who were diagnosed with atherosclerotic cardiovascular disease (ASCVD) and enrolled in the Jordan SMuRF-less Study (ClinicalTrials.gov Identifier: NCT06199869) between 10 January and 20 August 2024. Participants were recruited from nine healthcare centers across Jordan, including one private academic institution, three Ministry of Health hospitals, three community-based facilities, and two university-affiliated hospitals.

**Figure 1:**
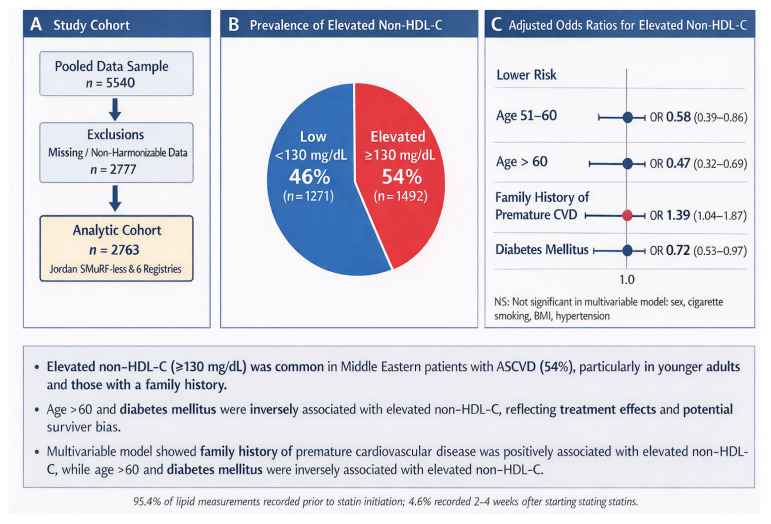
Summary of Study Findings.

The second source involved a retrospective, post hoc analysis of six well-established cardiovascular registries that had previously collected case data across the Middle East. The First: Jordan Percutaneous Coronary Intervention Registry (NCT01841346) [16], Surviving a Decade or More after Coronary Revascularization Study (NCT03491722) [16], Jordan Atrial Fibrillation Study (NCT03917992) [1], Jordan COVID-19 Pandemic Acute Cardiovascular Events Study (NCT04368637) [1], and Study of Novel and Classical Risk Factors in Young Middle Eastern Women with ASCVD (NCT04975503) [1]. Data were collected using standard case-report forms, completed by trained research assistants to ensure methodological consistency and data quality. Variables collected were demographic and anthropometric data, medical histories, established and novel cardiovascular risk factors (modifiable and non-modifiable), comorbidities, secondary prevention medications, and survival outcomes at one year post-event. Figure 1 summarizes study design.

### 2.2. Study Population and Cohort Attrition

The initial pooled sample included 5540 participants across the seven contributing registries [17,18]. After applying predefined inclusion and exclusion criteria and harmonizing variables across sources, 2763 participants had complete serum lipid measurements and essential covariates required for analysis. Participants were excluded primarily due to missing lipid profiles at enrollment or non-harmonizable/missing key variables required for cross-registry standardization.

### 2.3. Inclusion and Classification of Risk Factors

The included participants were those with a clinically diagnosed ASCVD, which was verified by documented evidence for coronary artery disease (CAD), ischemic stroke, peripheral artery disease, or carotid artery atherosclerosis. CAD were participants who had stable angina that was chronic, acute coronary syndromes (ACS) with STEMI and non-STEMI presentations, and also participants detected on coronary computed tomography angiography (CCTA). According to the number of standard modifiable cardiovascular risk factors (SMuRFs), participants were classified into three groups: those with no SMuRFs, one or two SMuRFs, and three or more SMuRFs.

### 2.4. Definition of Standard Modifiable Risk Factors (SMuRFs)

SMuRFs were treated as binary variables and defined using standardized diagnostic benchmarks informed by prior studies [18,19,20]. Dyslipidemia was identified through previous diagnosis, current lipid-lowering therapy, or high LDL-C levels beyond guideline-recommended thresholds. Type 2 diabetes mellitus (T2D) was identified based on medical records, the use of glucose-lowering agents, or HbA1c levels of 6.5% or higher. Hypertension (HTN) was defined by an existing diagnosis, antihypertensive treatment, or newly recorded elevated systolic blood pressure (≥140 mmHg) and/or diastolic blood pressure (≥90 mmHg) across multiple inpatient readings. Smoking status was considered positive if regular tobacco use was reported within the 12 months prior to enrollment.

The study also evaluated a traditional but non-SMuRF risk factor, family history of early-onset cardiovascular disease. A positive family history was identified when a first-degree male relative had a cardiovascular event before age 55 or a female relative before age 65. All lipid measurements in this study were reported using a consistent and clinically standard unit system. HDL-C, triglycerides, and non-HDL-C were expressed in mg/dL. At the same time, the total cholesterol-to-HDL-C (TC/HDL-C) ratio was treated as a unitless variable, as it represents the ratio of two measurements expressed in the same units. Metabolic syndrome was defined as the presence of three or more of the following criteria: hypertension, obesity, low HDL-C levels (<40 mg/dL for men and <50 mg/dL for women), and elevated triglycerides (>150 mg/dL). The TC/HDL-C ratio was categorized as follows: <3.5, indicating low risk; 3.5–5.0, indicating moderate risk; and >5.0, indicating high risk [10]. These classification thresholds are supported by established evidence and are consistent with international guidelines for cardiovascular risk evaluation. The ratio reflects the interplay between atherogenic and protective lipoprotein fractions, with higher values indicating an increased risk of atherosclerotic cardiovascular disease. Both the Framingham Heart Study and the European Society of Cardiology (ESC) have identified the TC/HDL-C ratio as a robust, independent predictor of coronary heart disease, recommending a ratio below 3.5 to minimize cardiovascular risk [10,21,22]. Ratios exceeding 5.0 are indicative of significantly heightened risk and are widely adopted in clinical risk assessment and lipid management protocols.

Finally, in Jordan, the SMuRF-less Study, non-high-density lipoprotein cholesterol (non-HDL-C) was calculated as total cholesterol minus HDL-C. Consistent with current lipid management guidelines, non-HDL-C was categorized as <130 mg/dL or ≥130 mg/dL, corresponding to commonly accepted thresholds for cardiovascular risk stratification [23,24].

### 2.5. Ethical Considerations

This study was conducted in accordance with the ethical principles of the Declaration of Helsinki and was approved by the Institutional Review Board/Independent Ethics Committee of Istishari Hospital, Amman, Jordan. The study protocol was registered on ClinicalTrials.gov (Identifier: NCT06199869).

The data analyzed were obtained from a pooled database of 5540 patients enrolled in previously published cardiovascular studies, all registered on ClinicalTrials.gov and conducted by the same investigative group. For the prospective cohort (Jordan SMuRF-less Study), all participants provided written informed consent prior to enrollment. For the retrospective registries, informed consent was obtained at the time of enrollment in each original study, as documented in their respective approved protocols.

For the current pooled analysis, the Institutional Review Board granted a waiver of additional patient consent because the analysis involved secondary use of fully de-identified data from previously completed studies under the custodianship of the same authors, posed minimal risk to participants, and re-contacting participants was deemed impracticable.

### 2.6. Data Harmonization

Variables from the prospective cohort and the six cardiovascular registries were harmonized prior to pooling. Standardized case report forms, aligned with international guidelines, ensured consistent definitions for ASCVD diagnosis, SMuRF classification, lipid measures, and comorbidities. Harmonization was conducted retrospectively where needed, and any variables with inconsistent definitions across sources were excluded from pooled analyses. Participants with non-harmonizable or missing essential variables (e.g., lipid profile components, key covariates) were excluded during harmonization, reducing the final analytic cohort from 5540 to 2763 individuals.

### 2.7. Lipid Measurements and Statin Therapy

Most lipid measurements were obtained prior to initiation of lipid-lowering therapy (95.4%), while the remaining measurements were collected 2–4 weeks after starting statin treatment. Statin intensity was not reported because treatment decisions, including dose and intensity, were not standardized and were left to the discretion of the treating physicians, resulting in substantial variability that could not be reliably categorized for analysis.

### 2.8. Statistical Analysis

Descriptive statistics were used to summarize demographic, clinical, and lipid profile characteristics. Categorical data were presented as frequencies and percentages, while continuous data were reported as means with standard deviations (SD). Group differences, such as comparisons between participants with low versus high non-HDL cholesterol or by total cholesterol/HDL ratio, were analyzed using chi-square tests for categorical variables and either independent-samples t-tests or one-way ANOVA for continuous variables, depending on the number of groups.

Non-HDL cholesterol (non-HDL-C) was categorized according to established clinical thresholds: “low” < 130 mg/dL and “high” ≥ 130 mg/dL, in accordance with current lipid management guidelines (e.g., AHA/ACC, ESC).

Multivariable logistic regression was used to identify factors independently associated with high non-HDL cholesterol levels. Covariates included in the model were age group, sex, BMI, smoking status, diabetes, hypertension, heart failure, chronic kidney disease (CKD), statin therapy, and family history of early-onset cardiovascular disease. Findings were reported as odds ratios (ORs) with 95% confidence intervals (CIs) and *p*-values. Missing values were not imputed to avoid potential biases that might arise from imputation assumptions. Potential confounders were selected a priori, and the final models included age, sex, smoking status, body mass index, hypertension, diabetes mellitus, and family history of premature cardiovascular disease. Physical activity, socioeconomic status, and dietary factors were excluded due to high missingness (>30%), inconsistent measurement across sites, and their potential role as intermediates. Missing data for included covariates were <5%, so complete-case analysis was used, and analytic sample sizes are reported with the regression results. Because missingness was minimal and non-systematic, multiple imputation was not performed. A *p*-value of <0.05 was considered statistically significant. All statistical analyses were conducted using IBM SPSS Statistics 27.

## 3. Results

### 3.1. Characteristics of the Entire Study Sample

A total of 2763 patients with ASCVD were analyzed; most were male (75%), and ages were fairly evenly distributed, with 39.1% older than 60 years. Nearly all (95%) had less than a bachelor’s degree. BMI patterns reflected a predominantly overweight and obese cohort (42.4% and 36.5%, respectively). Many participants reported a family history of premature CVD (42.7%) or current cigarette smoking (48.2%). Comorbid conditions were common, including hypertension (57.1%), dyslipidemia (83.9%), diabetes (53.7%), chronic kidney disease (7.5%), and heart failure (20.2%) (Table 1).

### 3.2. Patient Characteristics by Non-HDL Cholesterol

Of the 2763 patients, 1271 (46%) had low non-HDL cholesterol levels, and 1492 (54%) had elevated levels. Compared with those with low non-HDL cholesterol, patients with elevated levels were more likely to be younger (≤50 years: 36.3% vs. 22.9%, *p* < 0.001) and to report a family history of premature CVD (45.8% vs. 39.2%, *p* < 0.001). Cigarette smoking was also more common in the elevated non-HDL group (52.6% vs. 43.1%, *p* < 0.001). Conversely, comorbid conditions, including hypertension, diabetes, chronic kidney disease, and heart failure, were more prevalent among patients with low non-HDL levels. Dyslipidemia was markedly more frequent in those with elevated non-HDL cholesterol (90.8% vs. 75.8%, *p* < 0.001) (Table 2).

### 3.3. Patient Characteristics by Total Cholesterol/HDL Ratio

Stratification by total cholesterol/HDL ratio revealed progressive trends across categories. Patients in the elevated-risk group (>5.0) were more often male (81.8% vs. 62.9% in the low-risk group, *p* < 0.001), younger (≤50 years: 38.4% vs. 21.4%, *p* < 0.001), and more likely to smoke (59.7% vs. 35.3%, *p* < 0.001). A family history of premature CVD was also more common in the elevated-risk group compared to that in the low-risk group (46.9% vs. 37.8%, *p* = 0.001). In contrast, hypertension, chronic kidney disease, and heart failure were more frequent in the low-risk group. Dyslipidemia increased significantly across categories, affecting 88.6% in the elevated-risk group (*p* < 0.001) (Table 3).

### 3.4. Factors Associated with Elevated Non-HDL Cholesterol

In multivariate analysis, older age was independently associated with lower odds of elevated non-HDL cholesterol (51–60 years: OR 0.58, 95% CI 0.39–0.86; >60 years: OR 0.47, 95% CI 0.32–0.69; both vs. ≤50 years). A family history of premature CVD was also independently associated with elevated non-HDL cholesterol (OR 1.39, 95% CI 1.04–1.87). Conversely, diabetes mellitus was inversely associated with elevated non-HDL cholesterol (OR = 0.72; 95% CI, 0.53–0.97). No significant associations were observed for sex, residence, smoking status, BMI, or hypertension (Table 4).

A summary of the main findings—including the study flow, prevalence of elevated non-HDL-C, and adjusted odds ratios for key factors—is presented in Figure 1.

## 4. Discussion

This study of 2763 Middle Eastern patients with ASCVD highlights key demographic and clinical determinants of lipid abnormalities, emphasizing the roles of age, family history, smoking, and comorbidities in shaping profiles of non-HDL cholesterol and the total cholesterol/HDL ratio. Our findings extend prior regional evidence by showing that younger age and family history of premature CVD were most strongly associated with elevated non-HDL-C. At the same time, diabetes was inversely associated, reflecting complex treatment and survival dynamics.

### 4.1. Age and Lipid Profile

The predominance of younger adults (≤50 years) in the elevated non-HDL-C and high cholesterol/HDL ratio groups contrasts with patterns observed in Western cohorts, where dyslipidemia typically worsens with age. In our study, this younger age profile, along with a higher prevalence of family history of premature CVD among those with elevated non-HDL-C, may suggest a potential contribution from genetic factors, such as familial hypercholesterolemia (FH), as reported in other regional data [25,26,27]. However, because our dataset did not include genetic testing, dietary information, or formal FH diagnoses, these interpretations remain speculative and should be treated as hypotheses for future investigation. This finding underscores the need for targeted early screening programs and cascade testing for FH in Jordan, where underdiagnosis remains common [28,29].

In this cohort, younger patients (≤50 years) were more likely to have elevated non-HDL-C and higher total cholesterol/HDL-C ratios compared with older individuals. This age-related distribution differs from patterns commonly reported in Western populations, where lipid abnormalities often increase with advancing age [25,26,27]. However, given the cross-sectional design, these findings reflect associations rather than temporal trends and should not be interpreted as evidence of age-driven lipid progression. The higher prevalence of elevated non-HDL-C among younger participants, together with the greater frequency of family history of premature cardiovascular disease in this group, may suggest a potential contribution of inherited susceptibility. In particular, conditions such as familial hypercholesterolemia have been reported in the region [25,26,27]. However, our dataset did not include genetic testing, formal diagnostic criteria for familial hypercholesterolemia, or detailed longitudinal lipid histories. Therefore, any suggestion of genetic contribution remains speculative and should be considered hypothesis-generating rather than mechanistic. This observation underscores the need for improved recognition and cascade screening strategies for familial hypercholesterolemia in regional populations where underdiagnosis remains common [28,29].

Although survivorship bias may partly explain the lower non-HDL cholesterol levels observed in older patients, younger individuals with an adverse lipid profile may face similarly poor outcomes if early and intensive risk-factor management is not implemented. These findings highlight the importance of timely identification and aggressive lipid control in younger patients to reduce premature cardiovascular risk.

Conversely, older participants (>60 years) demonstrated lower odds of elevated non-HDL-C. Several non-mutually exclusive explanations may account for this association. First, survivorship bias may be present, as individuals with more severe dyslipidemia may have experienced earlier cardiovascular events or mortality and therefore may be underrepresented in older age groups [25,26]. Second, older adults are more likely to receive and adhere to lipid-lowering therapies, which could influence measured lipid levels [25,26]. Third, chronic illness, frailty, or other age-related metabolic changes may affect lipid concentrations independent of atherosclerotic burden [25,26]. These interpretations cannot be disentangled within a cross-sectional framework and require confirmation in longitudinal studies.

Overall, the observed age-related differences in lipid profiles should be interpreted cautiously. Rather than implying causal or biological mechanisms, these findings highlight demographic patterns that warrant further investigation using prospective designs with standardized treatment documentation and genetic characterization.

### 4.2. Smoking and Lipid Profiles

In univariate analyses, smoking was significantly more prevalent among patients with elevated non-HDL-C and higher total cholesterol/HDL-C ratios. This finding is consistent with established evidence that tobacco exposure is associated with lower HDL-C levels and higher triglycerides through oxidative stress, systemic inflammation, and endothelial dysfunction [30,31].

However, the association between smoking and elevated non-HDL-C was not maintained in multivariable models. This attenuation likely reflects confounding by other cardiometabolic factors included in the adjusted analysis, particularly age and diabetes, which are closely interrelated with both smoking behavior and lipid profiles. In addition, shared variance among covariates and potential collinearity may have reduced the independent contribution of smoking in the fully adjusted model.

Treatment-related factors may also partially explain this discrepancy. Patients with established ASCVD who smoke may be more likely to receive intensive lipid-lowering therapy or closer clinical monitoring, which could influence measured lipid levels independent of smoking status. Because statin intensity and adherence were not uniformly documented, the extent of this effect cannot be directly quantified.

Therefore, the absence of an independent association in multivariable analysis should not be interpreted as evidence that smoking lacks biological relevance to lipid metabolism. Rather, within this cross-sectional framework, smoking appears to cluster with other high-risk characteristics, and its independent statistical contribution becomes attenuated after adjustment. Nonetheless, given its high prevalence—particularly among men in this cohort—smoking cessation remains a critical component of cardiovascular risk reduction strategies in the region [32,33].

### 4.3. Diabetes and the Paradox of Lower Non-HDL-C

Unexpectedly, diabetes mellitus was inversely associated with elevated non-HDL-C after adjustment. A similar paradox has been observed in other studies, where patients with diabetes or multiple comorbidities exhibit lower lipid levels due to intensive treatment or disease-related metabolic changes [25,26]. Reverse causation may also contribute, as chronic illness and frailty can reduce cholesterol levels, potentially masking the actual risk. This highlights a key limitation of relying solely on lipid levels for risk assessment in complex patients, underscoring the need for a more comprehensive approach that integrates glycemic control, inflammation, and renal function.

### 4.4. Residual Risk and Non-HDL-C Superiority

Both non-HDL-C and the cholesterol/HDL ratio proved valuable in stratifying risk, consistent with global evidence that these measures better capture atherogenic burden than LDL-C alone [34,35,36]. Non-HDL-C includes remnant lipoproteins that often remain high despite statin therapy and contribute to residual cardiovascular risk [6,37]. The high prevalence of dyslipidemia (90.8%) among patients with elevated non-HDL-C in our cohort underscores the need for intensified treatment strategies targeting multiple lipid fractions.

Recent evidence underscores the variability in how hypercholesterolemia is integrated into cardiovascular risk assessment and highlights the utility of emerging biomarkers. Measures such as residual cholesterol, inflammatory markers, and polygenic risk scores help identify residual risk despite controlled LDL-C or non-HDL-C levels. Early life lipid exposure remains a key concern, accelerating atherosclerosis and elevating lifetime risk even with later treatment. Additionally, coronary artery calcium (CAC) scoring serves as a strong negative risk marker in severe hypercholesterolemia, guiding decisions on the need for pharmacologic therapy [38]. Integrating such biomarkers and imaging techniques into clinical practice could provide a more comprehensive and individualized assessment of residual risk, especially in Middle Eastern populations where early-onset dyslipidemia and familial hypercholesterolemia (FH) are highly prevalent.

Our findings carry important implications for Middle Eastern and global healthcare systems. First, early screening for dyslipidemia should be prioritized, particularly among younger adults with a family history of premature CVD. Second, therapeutic strategies must extend beyond LDL-C to address non-HDL-C and remnant cholesterol, in line with emerging international guidelines [22,23,39]. Third, addressing lifestyle determinants such as smoking, obesity, and physical inactivity remains essential, given their synergistic effects on lipid abnormalities [40,41,42]. Finally, policy-level interventions should integrate genetic risk assessment and promote culturally sensitive prevention programs to reduce the premature burden of ASCVD.

These findings highlight the critical need for a proactive, prevention-focused approach to cardiovascular care in Middle Eastern populations. Elevated non-HDL-C and cholesterol/HDL ratios were strongly associated with younger age, family history of premature CVD, and smoking, while diabetes appeared inversely linked due to treatment and survival effects. Younger patients with these risk factors may require earlier and more aggressive lipid monitoring, as their absolute risk may be underestimated using conventional age-based screening thresholds. Family history of premature CVD, in particular, represents a strong, non-modifiable risk factor that should trigger cascade screening and consideration of genetic counseling for familial hypercholesterolemia. Understanding these effect sizes enables clinicians to stratify patients more effectively, prioritize high-risk subgroups, and implement targeted preventive interventions, rather than relying solely on standard lipid cutoffs. Comprehensive management strategies should extend beyond LDL-C to include non-HDL-C, residual risk markers, and lifestyle interventions, while integrating genetic risk assessment, culturally tailored public health programs, and individualized therapeutic strategies. Public health measures should also actively encourage healthier behaviors and facilitate early identification of inherited risks. By combining early detection, individualized treatment, and population-level prevention efforts, healthcare systems in the region can more effectively reduce the burden of premature ASCVD and improve long-term cardiovascular outcomes.

### 4.5. Strengths and Limitations

This study leverages a large, diverse cohort of 5540 patients drawn from multiple Middle Eastern cardiovascular registries, enhancing statistical power and regional relevance. By focusing on populations at high risk for early-onset metabolic disease, it addresses an important gap in global cardiovascular research. The evaluation of non-HDL cholesterol and lipid ratios provides a broader assessment of atherogenic burden beyond LDL-C alone, aligning with current AHA/ACC and ESC recommendations. Subgroup analyses by age, sex, comorbidities, and lifestyle factors add clinical depth and highlight potential areas for targeted intervention, including the possibility of underrecognized familial hypercholesterolemia.

However, the cross-sectional nature of the study precludes establishing causality or temporal relationships. Selection bias may arise from the hospital-based sampling frame, whereas survivorship and reverse causation biases cannot be excluded. The lack of genetic and detailed lifestyle data, variability in data collection across registries, and incomplete information on medication adherence or dosing further limit interpretability.

The cross-sectional design precludes causal inference and limits interpretation to associations. Pooling prospective and retrospective data may have introduced heterogeneity in enrollment, disease severity, and treatment at the time of lipid measurement. Survivorship bias, particularly among older individuals, may also have influenced the lower prevalence of elevated non-HDL cholesterol observed in those with multiple comorbidities. Accordingly, the inverse associations with age, diabetes, and heart failure should be interpreted cautiously as hypothesis-generating findings rather than evidence of causality

An important limitation of this study is the incomplete documentation of lipid-lowering therapies, including statin use, treatment intensity, timing of initiation, adjunctive agents (e.g., ezetimibe, bempedoic acid, PCSK9 inhibitors), and other medications influencing lipid levels such as fibrates and thiazide diuretics. The absence of these data limits our ability to fully account for pharmacologic effects on lipid parameters and may introduce residual confounding in the observed associations for non-HDL-C and lipid ratios. Additionally, the lack of longitudinal follow-up precludes assessment of cardiovascular outcomes across lipid strata, and the unavailability of other atherogenic indices (e.g., LDL/HDL, ApoB/ApoA1, non-HDL-C/HDL, LDL/ApoB, triglycerides/HDL) restricts a more comprehensive evaluation of residual atherogenic risk. Accordingly, the findings should be interpreted as exploratory and hypothesis-generating rather than definitive.

Furthermore, detailed information on statin intensity and the use of other lipid-modifying therapies (such as fibrates or ezetimibe) was not uniformly available across the contributing registries. This variability may have resulted in an underestimation of the true atherogenic lipid burden, particularly among patients already receiving lipid-lowering therapy, as non-HDL cholesterol and remnant lipoproteins may have been partially suppressed at the time of assessment. Conversely, the persistence of elevated non-HDL-C levels despite treatment may more accurately capture residual cardiometabolic risk in real-world clinical practice rather than reflect measurement limitations alone. The lack of standardized data on treatment intensity further constrains the ability to distinguish pharmacologic effects from underlying metabolic risk. Accordingly, non-HDL-C levels in this analysis should be interpreted as pragmatic indicators of residual lipid burden in routine care settings rather than as precise estimates of untreated lipid risk.

Lipid measurements in our dataset represent a mix of baseline and on-treatment values, as prior therapy use and adherence could not be consistently verified across registries. This heterogeneity may affect the evaluation of residual lipid burden, particularly in patients with established ASCVD.

Moreover, although we reference follow-up frameworks in some contributing registries, this study relies solely on surrogate lipid endpoints rather than adjudicated cardiovascular outcomes such as myocardial infarction, stroke, or cardiovascular mortality. Finally, a small proportion of lipid measurements (4.6%) were obtained after the initiation of lipid-lowering therapy, resulting in a mix of on- and off-treatment values.

While statin use at the time of measurement was included as a covariate in multivariable analyses, on-treatment values—particularly among older patients and those with diabetes, heart failure, or chronic kidney disease—may have attenuated non-HDL cholesterol levels. This introduces variability across participants, limits direct comparability of non-HDL-C values, and may partially explain the observed inverse associations with age and diabetes, which should therefore be interpreted with caution.

Although the pathophysiology that makes non-HDL-C a useful atherogenic marker is universal, the observed distributions and clinical implications in our pooled Middle Eastern cohort may not generalize directly to other regions. In the Middle East and North Africa, rising dyslipidemia, high obesity rates, and distinct patterns of tobacco use have been reported, and regional registries document suboptimal lipid control despite high dyslipidemia prevalence, all of which shape the local non-HDL-C burden [43]. By contrast, many countries in Europe show higher statin utilization and more established secondary-prevention programs, which can lower population non-HDL-C and change the magnitude of associations with outcomes (statin utilization is substantially higher in Europe and North America than in MENA and East Asia) [44]. In parts of East Asia (for example, China), dyslipidemia prevalence and treatment patterns differ again—awareness and treatment rates are often lower than Western countries despite large absolute burdens—so observed thresholds and residual-risk relationships may diverge [45]. Genetic factors such as higher regional estimates of familial hypercholesterolemia in some Arabian Gulf populations may further amplify early-onset atherogenic burden and limit direct transferability of absolute thresholds [46].

## 5. Conclusions

This study of 2763 Middle Eastern patients with ASCVD demonstrates that elevated non-HDL-C and cholesterol/HDL ratios are most strongly associated with younger age, family history of premature CVD, and smoking. In contrast, diabetes and other comorbidities showed an inverse relationship likely reflecting treatment intensity and survival bias. These findings contrast with Western cohorts, where dyslipidemia typically rises with age, and highlight an earlier onset of atherogenic risk in Middle Eastern populations. Importantly, our results underscore the clinical superiority of non-HDL-C and cholesterol ratios over LDL-C alone in capturing atherogenic burden and residual risk, including the contribution of remnant cholesterol.

From a public health perspective, these findings support prioritizing earlier screening for dyslipidemia, integrating cascade testing for familial hypercholesterolemia, and expanding culturally tailored prevention strategies such as smoking cessation, obesity reduction, and promotion of physical activity. At the clinical level, regional guidelines should be updated to explicitly incorporate non-HDL-C and residual risk markers as treatment targets alongside LDL-C. Overall, improved lipid control, earlier risk detection, and personalized interventions, particularly in younger adults and those with genetic predisposition, are crucial to reducing the disproportionately high burden of ASCVD in the Middle East.

## Figures and Tables

**Table 1:** Baseline characteristics of the entire study sample (*n* = 2763).

Characteristic	*n* (%)
**Sex**	
Male	2073 (75.0%)
Female	690 (25.0%)
**Age**	
≤50	832 (30.1%)
51–60	851 (30.8%)
>60	1080 (39.1%)
**Education**	
<Bachelor	786 (95.0%)
≥Bachelor	41 (5.0%)
**Body Mass Index**	
Normal	419 (21.1%)
Overweight	844 (42.4%)
Obesity	727 (36.5%)
**Family history of premature CVD**	1181 (42.7%)
**Cigarette smoker**	1333 (48.2%)
**Comorbidities**	
Hypertension	1577 (57.1%)
Dyslipidemia	2317 (83.9%)
Diabetes mellitus	1485 (53.7%)
Chronic Kidney Disease	138 (7.5%)
Heart failure	403 (20.2%)

CVD, Cardiovascular Disease.

**Table 2:** Characteristics stratified by non-HDL-C categories (*n* = 2763).

Characteristic	Low (<130 mg/dL) *n* (%)	Elevated (≥130 mg/dL) *n* (%)	*p*-Value
**Sex**			0.121
Male	936 (73.6%)	1137 (76.2%)	
Female	335 (26.4%)	355 (23.8%)	
**Age**			<0.001
≤50	291 (22.9%)	541 (36.3%)	
51–60	350 (27.5%)	501 (33.6%)	
>60	630 (49.6%)	450 (30.2%)	
**Education**			0.055
<Bachelor	464 (96.3%)	322 (93.3%)	
≥Bachelor	18 (3.7%)	23 (6.7%)	
**Body Mass Index**			0.624
Normal	203 (20.4%)	216 (21.7%)	
Overweight	419 (42.1%)	425 (42.7%)	
Obesity	373 (37.5%)	354 (35.6%)	
**Family history of premature CVD**	498 (39.2%)	683 (45.8%)	<0.001
**Cigarette smoker**	548 (43.1%)	785 (52.6%)	<0.001
**Comorbidities**			
Hypertension	816 (64.2%)	761 (51.0%)	<0.001
Dyslipidemia	963 (75.8%)	1354 (90.8%)	<0.001
Diabetes mellitus	732 (57.6%)	753 (50.5%)	<0.001
Chronic Kidney Disease	92 (9.8%)	46 (5.0%)	<0.001
Heart failure	249 (25.0%)	154 (15.4%)	<0.001

Non-HDL cholesterol categories were defined as Low (<130 mg/dL) and Elevated (≥130 mg/dL). CVD = Cardiovascular Disease.

**Table 3:** Characteristics stratified by TC/HDL-C ratio categories * (*n* = 2763).

Characteristic	Low (<3.5) *n* (%)	Moderate (3.5–5.0) *n* (%)	Elevated (>5.0) *n* (%)	*p*-Value
**Sex**				<0.001
Male	376 (62.9%)	765 (74.6%)	932 (81.8%)	
Female	222 (37.1%)	261 (25.4%)	207 (18.2%)	
**Age**				<0.001
≤50	128 (21.4%)	267 (26.0%)	437 (38.4%)	
51–60	127 (21.2%)	334 (32.6%)	390 (34.2%)	
>60	343 (57.4%)	425 (41.4%)	312 (27.4%)	
**Education**				0.179
<Bachelor	279 (96.5%)	295 (95.2%)	212 (93.0%)	
≥Bachelor	10 (3.5%)	15 (4.8%)	16 (7.0%)	
**Body Mass Index**				0.871
Normal	107 (22.1%)	158 (21.2%)	154 (20.3%)	
Overweight	196 (40.4%)	320 (42.8%)	328 (43.3%)	
Obesity	182 (37.5%)	269 (36.0%)	276 (36.4%)	
**Family history of premature CVD**	226 (37.8%)	421 (41.0%)	534 (46.9%)	0.001
**Cigarette smoker**	211 (35.3%)	442 (43.1%)	680 (59.7%)	<0.001
**Comorbidities**				
Hypertension	386 (64.5%)	586 (57.1%)	605 (53.1%)	<0.001
Dyslipidemia	455 (76.1%)	853 (83.1%)	1009 (88.6%)	<0.001
Diabetes mellitus	334 (55.9%)	545 (53.1%)	606 (53.2%)	0.506
Chronic Kidney Disease	46 (10.4%)	53 (7.6%)	39 (5.5%)	0.008
Heart failure	126 (26.0%)	150 (20.1%)	127 (16.7%)	<0.001

* Cholesterol ratio categories were defined as Low risk < 3.5, Moderate risk 3.5–5.0, and Elevated risk > 5.0. CVD = Cardiovascular Disease.

**Table 4:** Multivariate Analysis of Factors Associated with elevated Non-HDL Cholesterol in Middle Eastern Patients with ASCVD.

	OR	95% Confidence Interval		*p*-Value
Age				
≤50	1			
51–60	0.58	0.39	0.86	0.007
>60	0.47	0.32	0.69	<0.001
Sex (Female vs. Male)	1.33	0.95	1.86	0.098
Residence (Rural vs. urban)	0.72	0.50	1.02	0.066
Family history of premature CVD	1.39	1.04	1.87	0.027
Cigarette smoking	0.97	0.69	1.35	0.845
Body Mass Index				
Normal	1			
Overweight	1.29	0.87	1.91	0.213
Obesity	1.02	0.69	1.52	0.903
Hypertension	0.88	0.64	1.21	0.432
Diabetes Mellitus	0.72	0.53	0.97	0.033

## Data Availability

The raw data supporting the conclusions of this article will be made available by the authors on request.
